# The German Version of the Dutch Eating Behavior Questionnaire: Psychometric Properties, Measurement Invariance, and Population-Based Norms

**DOI:** 10.1371/journal.pone.0162510

**Published:** 2016-09-22

**Authors:** Michaela Nagl, Anja Hilbert, Martina de Zwaan, Elmar Braehler, Anette Kersting

**Affiliations:** 1 Department of Psychosomatic Medicine and Psychotherapy, University of Leipzig, Leipzig, Germany; 2 Integrated Research and Treatment Center Adiposity Diseases, University of Leipzig Medical Center, Leipzig, Germany; 3 Department of Medical Psychology and Medical Sociology, University of Leipzig, Leipzig, Germany; 4 Department of Psychosomatic Medicine and Psychotherapy, Hannover Medical School, Hannover, Germany; 5 Department of Psychosomatic Medicine and Psychotherapy, University Medical Center of the Johannes Gutenberg University Mainz, Mainz, Germany; Hospital Universitario de la Princesa, SPAIN

## Abstract

The Dutch Eating Behavior Questionnaire is an internationally widely used instrument assessing different eating styles that may contribute to weight gain and overweight: emotional eating, external eating, and restraint. This study aimed to evaluate the psychometric properties of the 30-item German version of the DEBQ including its measurement invariance across gender, age, and BMI-status in a representative German population sample. Furthermore, we examined the distribution of eating styles in the general population and provide population-based norms for DEBQ scales. A representative sample of the German general population (N = 2513, age ≥ 14 years) was assessed with the German version of the DEBQ along with information on sociodemographic characteristics and body weight and height. The German version of the DEQB demonstrates good item characteristics and reliability (restraint: α = .92, emotional eating: α = .94, external eating: α = .89). The 3-factor structure of the DEBQ could be replicated in exploratory and confirmatory factor analyses and results of multi-group confirmatory factor analyses supported its metric and scalar measurement invariance across gender, age, and BMI-status. External eating was the most prevalent eating style in the German general population. Women scored higher on emotional and restrained eating scales than men, and overweight individuals scored higher in all three eating styles compared to normal weight individuals. Small differences across age were found for external eating. Norms were provided according to gender, age, and BMI-status. Our findings suggest that the German version of the DEBQ has good reliability and construct validity, and is suitable to reliably measure eating styles across age, gender, and BMI-status. Furthermore, the results demonstrate a considerable variation of eating styles across gender and BMI-status.

## Introduction

Overweight and obesity are recognized as major public health problems due to their dramatic physical health consequences and the associated economic burden [1–3]. Based on nationally representative data from the ‘German Health Interview and Examination Survey for Adults’ (DEGS 1, 2008–2011, Robert Koch Institute), the prevalence of overweight (body mass index (BMI) ≥ 25 kg/m^2^) in Germany was estimated at 53.0% for women and 67.1% for men. Of those, 23.9% (women) and 23.3% (men) were obese (BMI ≥ 30 kg/m^2^) [4].

Overweight and obesity are the result of a chronic positive energy balance. The pathways leading to this energy imbalance are highly complex including, for example, genetic, metabolic, neuroendocrine, socio-cultural, environmental, psychological and behavioral factors [5]. Among these factors, food intake and eating behavior play an important role in energy homeostasis. Considering the question of why some people tend to overeat and others do not, three psychological theories may explain the etiology of overeating [6]. The psychosomatic theory emphasizes the concept of emotional eating [7,8], i.e. eating in response to emotional arousal instead of internal signals of hunger or satiety, as an atypical response to distress. Usually, emotional arousal and stress are associated with a decrease of appetite and food intake, while it leads to increased food intake in emotional eaters. Externality theory [9] focuses on external eating, suggesting that some people are more sensitive to external food cues than others and are more likely to eat in response to these cues (e.g. the sight or smell of food) regardless of internal physiological states. Restraint theory focuses on dieting as a trigger for overeating [10]. Dieters suppress their feelings of hunger through cognitive control. If this cognitive control is disrupted, restrained eaters tend to eat more than non-dieters.

The Dutch Eating Behavior Questionnaire (DEBQ), developed by van Strien et al. [6], simultaneously measures all three types of overeating proposed in these theories. It consists of 33 items covering the domains *emotional eating* (13 items; e.g. ‘Do you have a desire to eat when you are irritated?’), *external eating* (10 items; ‘Do you eat more than usually when you see others eating?’), and *restraint* (10 items; ‘Do you deliberately eat less in order not to become heavier?’). All items are answered on a 5-point Likert scale ranging from 1 (‘never‘) to 5 (‘very often‘). The Dutch original version of the DEBQ has been found to have good psychometric properties regarding reliability, factor structure and predictive validity [11,12]. The DEBQ has been extensively used for the assessment of eating behavior and translated into many languages, amongst others English [13], Spanish [14], French [15], Italian [16], Turkish [17] and German [18]. It has been adapted for the assessment of eating behavior in children with either children [19,20] or parents as informants [21], and for the older and oldest population [22].

The German version of the DEBQ (‘Fragebogen zum Ernährungsverhalten [FEV-II] ‘) was developed by Grunert [18] on the basis of the English translation [13]. The German version incorporates two important modifications from the original Dutch and the English versions: 1) 3 items from the emotional eating subscale (eating when sb. lets you down; eating when frightened; eating when emotionally upset) were removed resulting in a total number 30 items (10 items per subscale), 2) while the items in the Dutch original and English version of the DEBQ were formulated as questions, items in the German version of the DEBQ were formulated as statements (e.g., emotional eating: ‘When I’m irritated, I have a desire to eat‘; external eating: ‘I eat more than usually when I see others eating.’; restraint: ‘I deliberately eat less in order not to become heavier’). The three subscales of the German 30-item version of the DEBQ have been found to have good internal consistency in different studies (0.81 ≤ α ≤ 0.94) [18,23–26]. Despite the extensive use of the German version of the DEBQ, little is known about its factor structure thus far. Results from an exploratory factor analysis (EFA) and multidimensional scaling in a convenience sample of 119 university students [18] revealed a four factor solution containing restraint, external eating and a two-dimensional emotional eating factor: eating in response to clearly labeled emotions (e.g., fear, anger) and eating in response to diffuse emotions (e.g., loneliness), which was also found for the Dutch original version [6]. In contrast, three factor solutions containing the three theoretical domains (restraint, emotional eating, external eating) were found for the English version [13] and other translations thereof [14]. Thus, the evidence with regard to the factorial validity of the DEBQ is somewhat controversial and the evaluation of the factor structure of the German version is limited to an exploratory approach applied in a small convenience sample of college students. Furthermore, item statistics for the German version of the DEBQ have not been systematically reported. Therefore, the first aim of our study was to evaluate the psychometric properties of the German version of the DEBQ, including item statistics, factorial structure using exploratory and confirmatory factor analysis (CFA), and internal consistency in a large population-based sample of the German general population.

Another important aspect of factor-analytic studies is to determine the measurement invariance across relevant subgroups. Studies examining DEBQ subscales as a function of gender and BMI-status consistently showed higher scores on DEBQ subscales among individuals with overweight compared to normal weight individuals [16,19,27–29]. Women were shown to score significantly higher than men on the restraint and emotional eating scale [13,16,22,30]. Research on the influence of age on the DEBQ subscales is limited indicating lower scores among older age groups on the emotional eating subscale [16,22]. Such between-group differences can only be interpreted when measurement invariance is given, i.e., if the same construct is measured in every subgroup [31,32]. Measurement invariance across gender, age, and BMI-status has rarely been assessed for the DEBQ [16,20,22]. It was confirmed for the 20-item DEBQ childrens’ version [20] and a 16-item French version adapted for the older population [22]. Dakanalis et al. [16] found evidence for measurement invariance of a 33-item Italian translation of the DEBQ across gender, age, and BMI-status. Measurement invariance has, however, not been examined for the 30-item German version of the DEBQ thus far. The second aim of our study was therefore to examine the measurement invariance of the German version of the DEBQ across gender, age, and BMI-status (underweight/normal weight, overweight, obesity) and to explore the distributions of eating behavior across gender, age, and BMI-status.

Furthermore, while norms for different subgroups are available for the Dutch and English versions of the DEBQ [6,13], norms for the German general population have not yet been reported. Therefore, the third aim of study was to provide population-based norms for the German version of the DEBQ based on a representative sample of the German general population.

## Materials and Methods

### Sampling

Between March and May 2015, a representative sample of the German population was recruited for a cross-sectional questionnaire survey. A three-stage random sampling procedure was conducted: 1) selection of 258 sample point areas using a random allocation procedure, (2) random selection of target households within the sample point areas through a random route procedure, (3) random selection of target persons within target households using a kish selection grid. Participants were in included in the study if they were 14 years or older, fluent in German, and provided written informed consent. The study was conducted according to the ethical standards of the Declaration of Helsinki and was approved by the Ethical Review Committee of the University of Leipzig.

### Field work and measures

The field work was conducted by an independent market and social research agency (USUMA, Berlin, Germany). Selected individuals were approached in-person by a trained interviewer. At maximum, four attempts were made to reach a target person. Potential participants were informed about the study and provided written informed consent including additional parental consent for minor participants. Interviewers collected sociodemographic information face-to-face. Afterwards, participants anonymously filled out a battery of self-report questionnaires, including the German version of the DEBQ [18] with 30 items on a five-point Likert scale (1 = ‘never‘, 5 = ‘very often’; for a detailed description of the German version of the DEBQ see introduction section). Body weight and height were assessed by self-report and the BMI was calculated using the formula: BMI = body weight (kg)/(body height (m^2^)). BMI was categorized into underweight/normal weight (BMI < 25 kg/m^2^), overweight (25 kg/m^2^ ≤ BMI < 30 kg/m^2^), and obesity (BMI ≥ 30 kg/m^2^) [33].

### Participants

A total of 4844 individuals were randomly sampled. Of these, 2576 agreed to participate and participated in the assessment (response rate: 53.2%). Reasons for non-participation were that households could not be reached (13.8%) or refused to participate (14.6%), that target persons could not be reached (2.0%), were out of town (0.6%) or incapacitated (0.4%), and that target persons refused to participate (15.4%). Overall, 63 cases had to be excluded from the analyses resulting in a final data set of 2513 individuals. A detailed overview of descriptive data is displayed in Table 1. Of the total study sample, 1394 (55.5%) were women and 1119 (44.5%) were men aged between 14 and 94 years (M = 48.79, SD = 18.11). Mean BMI was 25.78 kg/m^2^ (SD = 4.98).

**Table 1:** Sociodemographic characteristics of the normative sample.

		Total (N = 2513)	Women (N = 1394)	Men (N = 1119)
		N (%)	N (%)	N (%)
**Age, years**	≤ 24	277 (11.0)	137 (9.8)	140 (12.5)
	25–34	377 (15.0)	218 (15.6)	159 (14.2)
	35–44	374 (14.9)	208 (14.9)	166 (14.8)
	45–54	471 (18.7)	274 (19.7)	197 (17.6)
	55–64	461 (18.3)	245 (17.6)	216 (19.3)
	65–74	347 (13.8)	192 (13.8)	155 (13.9)
	≥ 75	206 (8.2)	120 (8.6)	86 (7.7)
**Residence**	Eastern Germany	501 (19.9)	268 (19.2)	233 (20.8)
	Western Germany	2012 (80.1)	1126 (80.8)	886 (79.2)
**Nationality**	German	2427 (96.6)	1346 (96.6)	1081 (96.6)
	Other	86 (3.4)	48 (3.4)	38 (3.4)
**Education**	< 12 years	1994 (79.3)	1116 (80.1)	878 (78.5)
	≥ 12 years	519 (20.7)	278 (19.9)	241 (21.5)
**Employment status**	Not unemployed	2377 (94.6)	1325 (95.1)	1052 (94.0)
	Unemployed	136 (5.4)	69 (4.9)	67 (6.0)
**Household income**	< 1250 EUR	427 (17.7)	291 (21.7)	136 (12.6)
	1250–2500 EUR	1070 (42.2)	591 (44.1)	479 (44.4)
	> 2500 EUR	922 (38.1)	457 (34.1)	465 (43.1)
**Living with a partner**	Yes	1375 (55.1)	710 (51.1)	665 (60.0)
	No	1122 (44.9)	679 (48.9)	443 (40.0)
**Body mass index**	Underweight / normal weight	1111(45.0)	683 (49.8)	428 (38.9)
	Overweight	967 (39.1)	455 (32.6)	512 (46.5)
	Obesity	393 (15.9)	233 (16.7)	160 (14.5)

Notes. Percentages are calculated from valid cases.

### Statistical analysis

All statistical analyses, if not otherwise indicated, were conducted using SPSS 20 and the significance level was set to α = .05.

#### Item analyses

We examined the item descriptives, percentages of missing values, item difficulties (in %) using the formula p_i_ = ((${\bar{x}}_{i}$−min(x_i_))/(max(x_i_)-min(x_i_))*100 (with ${\bar{x}}_{i}$ = mean of item i; min(x_i_) = minimal value on item i; max(x_i_) = maximum value on item i), and corrected item-total-correlations.

#### Internal factor structure

The internal factor structure of the DEBQ as an indicator of construct validity was evaluated by means of a split-half factor analysis approach. The total sample was randomly divided into two subsamples using the SPSS 20 random case selection procedure. In a first step, a Principal Axis Factor analysis (PAF) with VARIMAX rotation was conducted in the first split-half sample. Extraction criteria were eigenvalues > 1 in conjunction with a visual inspection of the scree plot. In a second step, a second PAF was conducted using a pre-determined number of factors according to the number of factors extracted in the first analysis and a criterion for factor loadings of ≥ 0.40. Then a CFA was performed in the second split-half sample testing the model obtained in the EFA using AMOS 23. The Standardized Root Mean Square Residual (SRMR) and the Root Mean Square Error of Approximation including the 90% confidence interval were used as absolute fit indices, and the Comparative Fit Index (CFI) and the Tucker-Lewis Index (TLI) were used as comparative fit indices. SRMR values < .08 indicate a good model fit. RMSEA values < .05 can be considered as good fit and values between .05 and .08 as acceptable fit. CFI and TLI values ≥ .90 indicate a good model fit [34,35].

#### Reliability

The internal consistency of the subscales was determined using Cronbach’s α. Mean inter-item correlations were calculated as an indicator of subscale homogeneity.

#### Measurement invariance across gender, age, and BMI-status

To test the measurement invariance across different subgroups, the sample was split by the variables of interest (gender, age, BMI-status). Firstly, the factorial model found in the total sample was fitted for different subgroups of age, gender, and BMI-status. Successive multi-group CFAs with nested models [31] were conducted to examine the measurement invariance across gender, age, and BMI-status using AMOS 23 [36]. In a first step, a multi-group baseline model (Model 0) was fitted. All subsequent models were compared to the baseline model. In a second step, we tested for metric invariance assuming the equivalence of factor loadings across groups (Model 1). In a third step, scalar invariance was tested by assuming equal factor loadings and equal item intercepts across groups (Model 2). As the Χ^2^-difference test is very sensitive to large sample sizes, Δ CFI was used as indicator of model equivalence. According to Cheung and Rensvold [32], changes ≥ .01 in the CFI between two nested models indicate a significant decrease in model fit and lead to a rejection of the constrained model.

#### Distribution of DEBQ subscales scores across gender, age, and BMI-status

Effects of gender, age and BMI on DEBQ subscale scores were assessed using multi- and univariate three-factorial (Gender*Age*BMI-status) analyses of variance (ANOVA) and post-hoc tests. Interaction effects were specified up to second-order (Gender*Age, Gender*BMI-status, Age*BMI-status). Results of univariate ANOVAs and post-hoc tests were only interpreted in case of significant higher-order effects. Partial η^2^ was calculated as estimation of effect size. Partial η^2^-values of 0.01 can be considered small and values of 0.06 and 0.14 medium and large effects, respectively.

#### Normative data

We calculated percentile ranks based on the DEBQ subscale raw scores for different subgroups.

## Results

### Item analyses

The item characteristics according to the numbering and subscale attribution of the German version of the DEBQ are displayed in Table 2. The percentage of missing values was low (≤ 0.7%) for all items with a mean percentage of missing items per subscale of 0.4% (SD = 0.07). About 37.0% (SD = 18.12) of the scores per domain fell into the lowest category (‘never‘). The emotional eating domain showed highest percentages of scores in the lowest category (M = 55.9%, SD = 6.58) while lowest percentages were found for the external eating domain (M = 19.8%, SD = 9.80). All items, except for three items of the external eating domain were positively skewed. Items of the restraint and external eating domain showed a negative kurtosis and items of the emotional eating domain showed a positive kurtosis. Item difficulties ranged between 13.3% and 50.0% indicating a low to medium probability of scores > 1 (‘never‘). Corrected item-total-correlations were moderate to high (0.48 ≤ *r*_it_ ≤ 0.80).

**Table 2:** Item and scale characteristics of the German version of the Dutch Eating Behavior Questionnaire (N = 2513).

									EFA (N_1_ = 1152)^a^^,^^b^			CFA (N_2_ = 1205)^b^
DEBQ	Item	M	SD	Skew-ness	Kurtosis	p_i_ (%)	r_it_	% missings	Factor I	Factor II	Factor III	λ_χ_
**Restraint**	5	2.40	1.22	0.39	-0.97	35.0	.70	0.4	.09	**.74**	.11	.73
	7	2.30	1.17	0.45	-0.83	32.5	.70	0.4	.16	**.71**	.16	.72
	10	2.30	1.18	0.48	-0.79	32.5	.77	0.3	.15	**.78**	.09	.80
	12	2.06	1.04	0.66	-0.41	26.5	.73	0.5	.27	**.70**	.17	.79
	13	2.20	1.14	0.60	-0.58	30.0	.80	0.4	.13	**.82**	.04	.84
	15	2.12	1.17	0.69	-0.62	28.0	.66	0.4	.10	**.70**	-.03	.67
	19	2.58	1.16	0.21	-0.86	39.5	.62	0.4	.00	**.69**	.02	.61
	21	2.09	1.09	0.77	-0.24	27.3	.67	0.2	.17	**.68**	.03	.71
	24	1.87	0.97	0.97	0.30	21.8	.72	0.3	.27	**.71**	.06	.77
	27	2.32	1.12	0.46	-0.60	33.0	.72	0.4	.10	**.74**	.16	.73
**Emotional Eating**	1 (CLE)	1.79	0.93	1.04	0.50	19.8	.79	0.6	**.76**	.15	.26	.82
	4 (CLE)	1.53	0.82	1.64	2.43	13.3	.75	0.5	**.74**	.12	.20	.80
	6 (CLE)	1.79	1.00	1.14	0.53	19.8	.80	0.4	**.75**	.17	.24	.83
	8 (DE)	1.86	1.01	0.97	0.11	21.5	.71	0.6	**.62**	.16	.34	.70
	9 (DE)	1.89	1.02	0.97	0.10	22.3	.71	0.5	**.61**	.17	.35	.70
	11 (CLE)	1.57	0.87	1.50	1.72	14.3	.79	0.4	**.79**	.18	.15	.83
	14 (CLE)	1.67	0.92	1.30	1.10	16.8	.81	0.5	**.80**	.20	.20	.85
	17 (CLE)	1.58	0.86	1.51	1.86	14.5	.79	0.4	**.79**	.15	.24	.82
	22 (DE)	1.72	0.96	1.21	0.65	18.0	.79	0.6	**.74**	.17	.25	.82
	30 (CLE)	1.57	0.86	1.47	1.63	14.3	.80	0.5	**.82**	.15	.21	.83
**External Eating**	2	2.39	1.00	0.25	-0.54	34.8	.62	0.5	.28	.05	**.59**	.65
	3	2.79	1.04	0.00	-0.56	44.8	.68	0.4	.14	.05	**.72**	.71
	16	2.51	1.03	0.14	-0.65	37.8	.60	0.4	.12	.20	**.63**	.60
	18	2.64	1.10	0.06	-0.76	41.0	.66	0.7	.21	.03	**.68**	.70
	20	3.00	1.09	-0.09	-0.68	50.0	.48	0.4	.10	.07	**.51**	.49
	23	2.18	1.04	0.50	-0.57	29.5	.68	0.5	.37	.10	**.64**	.74
	25	3.02	1.05	-0.17	-0.38	50.5	.65	0.3	.09	.06	**.71**	.68
	26	2.61	1.02	0.11	-0.57	40.3	.77	0.4	.27	.07	**.76**	.82
	28	2.55	1.02	0.15	-0.54	38.8	.64	0.4	.27	.09	**.64**	.67
	29	1.95	0.97	0.76	-0.18	23.8	.62	0.3	.39	.09	**.58**	.66

Notes. DEBQ, Dutch Eating Behavior Questionnaire; M, mean; SD, standard deviation; p_i_, item difficulty; r_it_, corrected item-total correlation; CLE, clearly labeled emotions; EFA: exploratory factor analysis; CFA: confirmatory factor analysis; DE, diffuse emotions; λ_χ_, standardized coefficients; n_1_/n_1,_ randomly selected subsamples

^a^Principal Axis Factor analysis with orthogonal VARIMAX rotation (extraction eigenvalues > 1 and scree test), highest load per item is bolded

^b^exploratory and confirmatory factor analyses were conducted for cases with complete DEBQ data (total N = 2357)

### Internal factor structure

The PAF conducted in the first randomly selected subsample (N_1_ = 1152) revealed three factors with eigenvalues > 1, which were confirmed by a visual inspection of the scree plot and a second PAF using a pre-determined number of three factors and factor loadings ≥ 0.40. The three factors explained 56.6% of the variance and replicated the three DEBQ subscales perfectly. The first factor (eigenvalue = 6.42) explained 21.4% of the variance and was composed of the 10 items from the emotional eating subscale. The 10 items of the restraint subscale loaded on the second factor (eigenvalue = 5.61) which explained 18.7% of the variance and the third factor (eigenvalue = 4.95, explained variance = 16.5%) was made up by the 10 items of the external eating subscale. All primary factor loadings were ≥ 0.51, however, two items (8 and 9) of the emotional eating subscale showed substantial cross-loadings (≥ 0.30) on the external eating domain and two items (23 and 29) of the external eating domain displayed similar cross-loadings on the emotional eating domain. Fit indices for the CFA in the second randomly selected subsample (N_2_ = 1205) are displayed in the upper part of Table 3. A three-factor model with correlated factors (restraint, emotional eating, external eating) and cross-loadings fixed to 0 revealed acceptable scores for the CFI, RMSEA and SRMR. Each item significantly loaded on the specified factor (all p < .001). The TLI was slightly out of the acceptable range (TLI = .895). Additional inspection of the modification indices (MIs) and standardized expected parameter changes (SEPCs) showed that this was a result of correlated unique variances of items 8 and 9, which both assess eating in response to diffuse emotions (eating when bored, eating when nothing to do). After allowing the unique variances of both items to correlate (three-factor model (a)), the model fit substantially increased, indicating a good model fit (Table 3). PFA factor loadings and CFA standardized coefficients are displayed in Table 2.

**Table 3:** Goodness-of-fit for the DEBQ three-factor model across different groups and measurement invariance across gender, age and BMI.

		CFI	TLI	RMSEA (90%CI)	SRMR	Δ CFI
Split half sample (N_2_ = 1205)	Three-factor model	.903	.895	.068 (.066-.071)	.054	—
	Three-factor model (a)	.937	.931	.055 (.053-.058)	.054	—
Gender^a^						
	Women (N = 1052)	.930	.924	.059 (.056-.061)	.057	—
	Men (N = 1305)	.920	.913	.061 (.058-.064)	.057	—
Multi-group analysis^a^						
	Model 0 (configural invariance)	.926	.919	.042 (.041-.044)	.057	—
	Model 1 (metric invariance)	.925	.921	.042 (.040-.043)	.059	.001
	Model 2 (scalar invariance)	.917	.916	.043 (.042-.044)	.060	.009
Age^a^						
	14–24 years (N = 258)	.893	.884	.074 (.068-.080)	.068	
	25–34 years (N = 360)	.914	.907	.065 (.060-.070)	.063	
	35–44 years (N = 348)	.928	.922	.062 (.057-.067)	.061	
	45–54 years (N = 438)	.903	.895	.070 (.066-.074)	.065	
	55–64 years (N = 435)	.924	.918	.060 (.056-.065)	.056	
	65–74 years (N = 328)	.913	.905	.066 (.061-.072)	.060	
	≥ 75 years (N = 190)	.890	.881	.069 (.062-.077)	.074	
Multi-group analysis^a^						
	Model 0 (configural invariance)	.904	.906	.025 (.024-.025)	.096	
	Model 1 (metric invariance)	.903	.906	.025 (.024-.025)	.095	.001
	Model 2 (scalar invariance)	.898	.907	.025 (.024-.025)	.095	.006
BMI^a^						
	Underweight/normal weight (N = 1038)	.929	.923	.057 (.055-.060)	.057	
	Overweight (N = 912)	.919	.912	.063 (.060-.066)	.055	
	Obesity (N = 369)	.912	.905	.066 (.062-.071)	.071	
Multi-group analysis^a^						
	Model 0 (configural invariance)	.922	.916	.035 (.034-.036)	.057	
	Model 1 (metric invariance)	.921	.918	.035 (.034-.036)	.058	.001
	Model 2 (scalar invariance)	.915	.916	.035 (.034-.036)	.058	.007

Notes. CFA = confirmatory factor analysis; CFI = comparative fit index; CI, confidence interval, RMSEA = Root Mean Square Error of Approximation; SRMR = standardized root mean square residual; three-factor model = correlated factors with cross-loadings fixed to 0; three-factor model (a) = correlated factors with cross-loadings fixed to 0 and correlation of unique variances between items 8 and 9 (diffuse emotions); Δ CFI, difference values between model 1/2 and model 0

^a^, three-factor model with correlation of unique variances between items 8 and 9 (diffuse emotions).

### Reliability

Cronbach’s α was .92 for the restraint scale, .94 for the emotional eating scale, and .89 for the external eating scale. Mean inter-item correlations slightly exceeded the ideal range of .2 to .4. They were .55 for the restraint subscale, .65 for the emotional eating subscale, and .46 for the external eating subscale. Significant positive correlations between subscales were found (restraint–external eating: r = .23; restraint–emotional eating: r = .36; emotional eating–external eating: r = .57; all p-values < .001).

### Measurement invariance across gender, age, and BMI-status

Table 3 shows the fit measures of the multi-group models for testing measurement invariance across gender, age, and BMI-status. In a first step, the same three-factor model (with correlated unique variance between items 8 and 9) which revealed a good fit in pervious CFA was calculated for both genders, the seven age groups and underweight/normal weight, overweight and obese individuals. Model fit was acceptable for all subgroups, with the exception of age groups 14–24 years and ≥ 75 years where the CLI and TLI were slightly below the acceptable range while all other fit indices scored above the cut-offs.

Measurement invariance was tested using multi-group comparisons with nested models. Considering gender, model 0 –assuming that the three-factor model fits for both groups with varying parameter values (configural invariance)–showed an adequate fit. The model fit for model 1 –assuming equal factor loadings (metric invariance) was nearly similar and Δ CFI was inferior to .01. Although the fit of model 2 –assuming equal factor loadings and equal item intercepts (scalar invariance)–showed a slightly worse fit than model 0, Δ CFI was still inferior to 0.01 suggesting that measurement invariance can be assumed across gender. Similar results were found for age and BMI-status (Δ CFI inferior to .01) indicating that the structure, factor loadings and item intercepts are invariant across age and BMI-status (Table 3).

### Distributions of eating behavior across gender, age, and BMI-status

Means and standard deviations for the DEBQ subscales in the total sample and across gender, age, and BMI-status are displayed in Table 4. In the total sample and all subgroups external eating was the most prevalent type of eating behavior, followed by restraint and emotional eating. Results from a three-factorial (Gender*Age*BMI-status) multivariate ANOVA revealed significant main effects for gender, age, and BMI-status and significant interaction effects for Age*BMI-status and Gender*BMI-status (gender: F(3, 2418) = 50.23; age: F(18, 7260) = 4.01; BMI-status F(6, 4868) = 21.06; Age*BMI-status: F(36, 7260) = 2.06; Gender*BMI-status: F(6, 4838) = 3.31, all p-values < .01). Univariate analyses revealed significant main effects of gender on the restraint and emotional eating subscales (0.01 ≤ η^2^ ≤ 0.04), significant main effects of age on the emotional eating and external eating subscales (0.01 ≤ η^2^ ≤ 0.02) and significant main effects of BMI-status on all DEBQ subscales (0.02 ≤ η^2^ ≤ 0.03) (all p-values < .05). Women showed significantly higher scores of restraint and emotional eating than men. Post-hoc test revealed that younger age groups showed significantly higher scores of emotional eating and external eating than older age groups. Individuals with overweight and obesity scored significantly higher on all DEBQ subscales compared to normal weight individuals. On the emotional and external eating subscales, individuals with obesity also showed significantly higher scores compared to individuals with overweight (Table 4). Univariate analyses also revealed significant interaction effects of Age*BMI-status on the emotional eating and external eating subscales (all p-values < .01). Effect sizes were, however, negligibly small (η^2^ ≤ 0.01), and the interaction effect may thus be attributable to the large sample size. No interaction effects were found for BMI-status and gender.

**Table 4:** Means, standard deviations and Cronbach’s α for DEBQ subscales in the total sample and across gender, age and BMI-status.

	Restraint				Emotional Eating				External Eating			
	M (SD)	α	F / post-hoc	Partial η^2^	M (SD)	α	F / post-hoc	Partial η^2^	M (SD)	α	F / post-hoc	Partial η^2^
Total sample (N = 2513)	2.23 (0.87)	.92			1.70 (0.76)	.94			2.57 (0.74)	.89		
Gender			105.69***	0.04			33.78***	0.01			1.13	0.001
Women (N = 1394)	2.42 (0.88)	.92			1.78 (0.80)	.95			2.55 (0.74)	.90		
Men (N = 1119)	1.97 (0.80)	.92			1.59 (0.68)	.94			2.58 (0.74)	.89		
Age, years			1.19	0.003			3.34*	0.008			9.64***	0.02
14–24 (N = 277)	2.11 (0.94)	.94	—		1.78 (0.80)	.94	a		2.71 (0.78)	.89	a, b, c	
25–34 (N = 377)	2.26 (0.92)	.93	—		1.69 (0.73)	.94			2.66 (0.72)	.89	d, e	
35–44 (N = 374)	2.16 (0.85)	.93	—		1.72 (0.79)	.95			2.65 (0.75)	.90	f, g	
45–54 (N = 471)	2.28 (0.84)	.92	—		1.75 (0.77)	.95			2.61 (0.74)	.90	h, i	
55–64 (N = 461)	2.29 (0.85)	.91	—		1.69 (0.76)	.95			2.51 (0.70)	.88	a, j	
65–74 (N = 374)	2.28 (0.86)	.92	—		1.62 (0.70)	.95			2.45 (0.74)	.91	b, d, f, h	
≥ 75 (N = 206)	2.08 (0.79)	.90	—		1.57 (0.70)	.94	a		2.29 (0.69)	.87	c, e, g, i, j	
Body mass index			41.06***	0.03			38.27***	0.03			23.98***	0.02
Underweight/normal weight (N = 1111)	2.08 (0.91)	.93	a, b		1.56 (0.64)	.93	a, b		2.46 (0.72)	.89	a, b	
Overweight (N = 967)	2.32 (0.84)	.92	a		1.71 (0.74)	.94	a, c		2.59 (0.72)	.89	a, c	
Obesity (N = 393)	2.40 (0.77)	.90	b		2.06 (0.94)	.96	b, c		2.80 (0.77)	.90	b, c	

Notes. M, mean; SD, standard deviation; underweight/normal weight, BMI < 25 kg/m^2^; overweight, 25 kg/m^2^ ≤ BMI < 30 kg/m^2^; obesity, BMI ≥ 30 kg/m^2^; F, F-value (main effect) from univariate analyses of variance (ANOVA), post-hoc; results from Bonferroni-corrected post-hoc subgroup comparisons

***, p < .001

*, p < .05

Cronbach’s α of the DEBQ subscales was assessed in every subgroup showing a good internal consistency across different groups (.88 ≤ α ≤ .96). In all subgroups, DEBQ subscales were significantly correlated (.13 ≤ r ≤ .61, all p-values < .05) except for the the restraint and external eating subscale among participants with obesity (r = .10, p = .056). Highest correlations were consistently found for the emotional and external eating subscale (.54 ≤ r ≤ .61, all p-values < .001).

### Normative data

Given the measurement invariance of the DEBQ subscales across gender, age and BMI-status and the significant main effects for gender, age and BMI but negligible interaction-effects between the variables, we calculated percentile ranks based on the DEBQ subscale raw scores for gender, age and BMI-status separately. Age-groups with homogeneous eating behavior were combined (S1–S3 Tables).

## Discussion

The DEBQ, originally developed by van Strien et al. [20], is a prominent questionnaire which is extensively being utilized internationally to assess three theoretically derived (psychosomatic theory, externality theory, restraint theory) types of eating behavior associated with weight gain and overweight: restraint, emotional eating, and external eating. Our study aimed to extend previous work on the 30-item German version of the DEBQ by examining its dimensional structure using an exploratory and confirmatory approach, by testing configural, metric and scalar measurement invariance across gender, age, and BMI-status, and by providing norm data based on a representative sample of the German general population. Furthermore, item descriptives and internal consistency were evaluated.

Item descriptives showed that the German version of the DEBQ was very well accepted by the participants as reflected by the very low percentage of missing values on DEBQ items (≤ 0.7%). Item difficulties were medium to high and items were mostly positively skewed supporting the questionnaire’s focus on atypical eating behaviors. Item difficulties and skewness were highest for emotional eating which is considered an atypical response to emotional distress [7,8] and lowest for external eating. Results for corrected item-total correlations and item homogeneity were satisfactory reflecting findings for the Dutch original version [6]. Cronbach’s α exceeded the critical value of .80 for all subscales indicating good internal consistency, which is in accordance with findings for the Dutch original version [6] and previous findings for the German version of the DEBQ, based on small convenience or clinical samples [18,23–26]. The internal consistency of the DEBQ subscales was equally high across genders, different age-groups and BMI-status, indicating that the German version of the DEBQ reliably measures eating behavior across different subgroups.

The construct validity of the German version of the DEBQ was examined using EFA and CFA. Results from the EFA indicated a three-factor solution which replicated the three theoretical domains restraint, emotional eating and external eating and explained 56.6% of the variance. The finding of substantial cross-loadings of items 8 (eating when nothing to do) and 9 (eating when bored) has also been observed for the original [6] and translated (e.g. English, French, Spanish) versions of the DEBQ [13–15], reflecting the partial inter-relatedness of the concepts of external and emotional eating [16]. The three-factor model showed an acceptable fit in a CFA confirming the three main theoretical factors of the original version [6] and the three-factor structure which has been reported for the English version of the DEBQ [13] and translations thereof [14–16]. The model fit substantially improved when allowing for unique variances of items 8 (eating when nothing to do) and 9 (eating when bored) to correlate. This modification is reasonable because both items assess eating in response to diffuse emotions while most other items loading on the factor assess eating in response to clearly labeled emotions. Furthermore, both items showed substantial cross-loadings on the external eating domain in EFA. Thus, the correlated unique variances of both items may resemble an underlying diffuse emotions or external eating latent construct. In the German translated version of the DEBQ the wording of both items is quite similar due to a rough translation of ‘restless‘ into ‘nothing to do‘ (item 8: ‘I have the desire to eat when I have nothing to do‘; item 9: ‘I have the desire to eat when I am bored or I have nothing to do‘), which may also contribute to the fact that both items also represent manifest variables for another latent concept which was not specified in the model.

Besides one study evaluating the measurement invariance across gender, age and BMI-status in an adult sample using a 33-item Italian translation of the DEBQ, our study is the first to systematically examine measurement invariance across these subgroups for the 30-item German version of the DEBQ in a large-scale study taking into account the entire life-span (14–94 years). Using multi-group comparisons of nested models, our results demonstrate configural, metric, and scalar measurement invariance across gender, age, and BMI-status (normal weight, overweight, obesity) indicating that the German version of the DEBQ validly measures restraint, emotional eating and external eating across different subgroups. However, considering the models for the youngest (14–24 years) and oldest (≥ 75 years) in the study, CFI and TLI scored slightly out of the acceptable range, while the RMSEA and the SRMR were still in the acceptable range, indicating that it may be of value to develop adapted versions of the DEBQ for the older and oldest population as it has been done by Bailly et al. [22] and adolescents.

The significant positive correlation between emotional and external eating found in the total sample and across all subgroups corroborates findings from previous studies [6,13–16]. The finding also corresponds with the theory that, although emotional eating and external eating are different concepts, both emotionality and food cues can operate together and promote eating behavior disregarding internal signals of hunger or satiety [6,16,19,20]. Furthermore, negative emotions or emotional distress may increase the awareness of the environment (e.g., the immediate food environment) and decrease the awareness of the self [37]. Contradictory to previous findings [13,16,19,20] where non-significant or even negative correlations were found between restrained eating and both emotional and external eating, we found lower but still significant positive correlations in the total sample and most subgroups. While the correlation between external eating and restraint was negligibly low, a medium-low relation was found between emotional eating and restraint indicating that at least for some individuals who consciously restrict their food intake emotional distress may be a factor leading to a disruption of restrictive cognitive control [6].

Considering the distributional analysis in a representative sample of the German population, external eating was found the most prevalent eating behavior in the total sample and across gender, age, and BMI-status, followed by restraint and emotional eating. Corroborating findings from previous studies [13,16,22,30,38] women showed higher levels of emotional eating and restraint. Higher scores of restraint among women reflect the fact that women are more likely than men to diet [16,22], which may be explained by higher levels of weight and shape concerns among women [39] and current standards for female beauty in the society [16,22]. Most prominent effects of age were found for external eating indicating higher levels of external eating among younger age groups compared to older age groups, which is in line with prior research [14,27]. Furthermore, BMI-status and eating behavior were significantly related, with individuals with obesity and overweight scoring significantly higher than normal weight individuals on all DEBQ subscales and individuals with obesity scoring significantly higher than overweight individuals on emotional and external eating subscales. This result supports the theoretical basis of the DEBQ. The finding that restrained eating is more frequent in overweight and obese individuals is in line with several previous studies [14,19,27,28] supporting the assumption that, paradoxically, restrained eating may be a risk factor for overconsumption and weight gain when cognitive self-control is undermined [10]. Based on these findings, population norms were provided for several subgroups according to gender, age and BMI-status.

A major strength of our study is the use of a large, population-based sample representative of the German general population with regard to gender and age. To the best of our knowledge, our study is the first to examine the factor structure of the German version of the DEBQ in a large sample using a confirmatory approach and to prove its measurement invariance across gender, age, and BMI-status. The provided population based norms will enhance the applicability of the German version of the DEBQ with regard to individual diagnostics. Major limitations of our study concern the fact that we did not collect data on retest-reliability and criterion validity, which means that two important other indicators of psychometric quality were not evaluated. The cross-sectional design of the study does not allow for conclusions about the direction of obtained associations (e.g., between eating behavior and overweight) and precludes the assessment of measurement invariance over time. Furthermore, the calculation of BMI was based on self-reported body weight and height. Although it has been shown that self-reported and objectively measured BMI are strongly correlated, BMI based on self-report tends to underestimate the real BMI due to a typical overvaluation of body height and an undervaluation of body weight in self-report [40]. The BMI-distribution in our study differs from the BMI-distribution found in the large representative ‘German Health Interview and Examination Survey for Adults’ (DEGS1 study, N = 7116) conducted by the Robert Koch Institute between 2008 and 2011 using objectively measured body weight and height. While in our study 49.3% women and 61.3% men were overweight or obese, the prevalence of overweight and obesity was estimated at 53.0% for women and 67.1% for men. The prevalence of obesity was 16.7% among women and 14.5% among men, while the DEGS study found prevalence figures of 23.3% and 23.9%, respectively [4]. This discrepancy may at least partly be attributed to the use of self-reported height and weight and limits the generalizability of our findings.

In conclusion, our study demonstrated that the 30-item German version of the DEBQ [18] has adequate psychometric properties and reliably measures eating behavior across age, gender, and BMI-status. Furthermore, our study indicated measurement invariance of the DEBQ across gender, age, and BMI-status supporting the assumption that the construct validity of the instrument is not influenced by these variables. The provided population-based norms can be applied for diagnostic purposes. Detailed information on different eating styles is of value for the prevention and treatment of overweight and obesity.

## Supporting Information

S1 Dataset Participant-level study data. (SAV)

S1 Table Gender-specific norm values fort the German version of the Dutch Eating Behavior Questionnaire (N = 2513). (DOCX)

S2 Table Age-specific norm values fort the German version of the Dutch Eating Behavior Questionnaire (N = 2513). (DOCX)

S3 Table BMI-specific norm values fort the German version of the Dutch Eating Behavior Questionnaire (N = 2513). (DOCX)
