# Supplementary material for: The German Version of the Dutch Eating Behavior Questionnaire: Psychometric Properties, Measurement Invariance, and Population-Based Norms
Source: PLoS One. 2016 Sep 22;11(9):e0162510. doi: 10.1371/journal.pone.0162510 (PMC5033316; doi:10.1371/journal.pone.0162510)
Supplement: S1 Table — (DOCX) [file pone.0162510.s002.docx]

**Table S1**. **Gender-specific norm values fort the German version of the Dutch Eating Behavior Questionnaire (N = 2513)**

| **Females (N = 1394)** | | | | | **Males (N = 1119)** | | | |
| --- | --- | --- | --- | --- | --- | --- | --- | --- |
| Percentile Rank | Restraint | Emotional Eating | External Eating | Percentile Rank | | Restraint | Emotional Eating | External Eating |
| 5 | - | - | 1.32 | 5 | | - | - | 1.39 |
| 10 | 1.20 | - | 1.60 | 10 | | - | - | 1.60 |
| 15 | 1.30 | - | 1.80 | 15 | | 1.10 | - | 1.80 |
| 20 | 1.50 | - | 1.90 | 20 | | 1.20 | - | 1.90 |
| 25 | 1.70 | 1.10 | 2.00 | 25 | | 1.30 | - | 2.00 |
| 30 | 1.90 | 1.20 | 2.19 | 30 | | 1.40 | 1.10 | 2.20 |
| 35 | 2.10 | 1.20 | 2.30 | 35 | | 1.50 | 1.10 | 2.30 |
| 40 | 2.20 | 1.30 | 2.40 | 40 | | 1.60 | 1.20 | 2.31 |
| 45 | 2.30 | 1.40 | 2.40 | 45 | | 1.80 | 1.20 | 2.45 |
| 50 | 2.45 | 1.50 | 2.50 | 50 | | 1.90 | 1.30 | 2.60 |
| 55 | 2.60 | 1.70 | 2.60 | 55 | | 2.00 | 1.40 | 2.70 |
| 60 | 2.70 | 1.90 | 2.70 | 60 | | 2.10 | 1.50 | 2.80 |
| 65 | 2.80 | 2.00 | 2.80 | 65 | | 2.30 | 1.70 | 2.90 |
| 70 | 2.90 | 2.10 | 2.90 | 70 | | 2.50 | 1.90 | 3.00 |
| 75 | 3.00 | 2.30 | 3.00 | 75 | | 2.60 | 2.00 | 3.10 |
| 80 | 3.20 | 2.50 | 3.20 | 80 | | 2.70 | 2.20 | 3.20 |
| 85 | 3.30 | 2.70 | 3.40 | 85 | | 2.90 | 2.40 | 3.30 |
| 90 | 3.50 | 3.00 | 3.50 | 90 | | 3.10 | 2.60 | 3.50 |
| 95 | 3.90 | 3.30 | 3.80 | 95 | | 3.40 | 2.90 | 3.90 |
| 99 | 4.60 | 4.21 | 4.40 | 99 | | 4.00 | 3.58 | 4.38 |
